# Tobacco smoke induced COPD/emphysema in the animal model—are we all on the same page?

**DOI:** 10.3389/fphys.2013.00091

**Published:** 2013-05-15

**Authors:** Maike Leberl, Adelheid Kratzer, Laimute Taraseviciene-Stewart

**Affiliations:** Division of Pulmonary Sciences and Critical Care Medicine, Department of Medicine, University of Colorado School of MedicineDenver, CO, USA

**Keywords:** second hand cigarette smoke, COPD, emphysema, inflammation, animal model, chamber, pulmonary hypertension, matrix degradation

## Abstract

Chronic Obstructive Pulmonary Disease (COPD) is one of the foremost causes of death worldwide. It is primarily caused by tobacco smoke, making it an easily preventable disease, but facilitated by genetic α-1 antitrypsin deficiency. In addition to active smokers, health problems also occur in people involuntarily exposed to second hand smoke (SHS). Currently, the relationship between SHS and COPD is not well established. Knowledge of pathogenic mechanisms is limited, thereby halting the advancement of new treatments for this socially and economically detrimental disease. Here, we attempt to summarize tobacco smoke studies undertaken in animal models, applying both mainstream (direct, nose only) and side stream (indirect, whole body) smoke exposures. This overview of 155 studies compares cellular and molecular mechanisms as well as proteolytic, inflammatory, and vasoreactive responses underlying COPD development. This is a difficult task, as listing of exposure parameters is limited for most experiments. We show that both mainstream and SHS studies largely present similar inflammatory cell populations dominated by macrophages as well as elevated chemokine/cytokine levels, such as TNF-α. Additionally, SHS, like mainstream smoke, has been shown to cause vascular remodeling and neutrophil elastase-mediated proteolytic matrix breakdown with failure to repair. Disease mechanisms and therapeutic interventions appear to coincide in both exposure scenarios. One of the more widely applied interventions, the anti-oxidant therapy, is successful for both mainstream and SHS. The comparison of direct with indirect smoke exposure studies in this review emphasizes that, even though there are many overlapping pathways, it is not conclusive that SHS is using exactly the same mechanisms as direct smoke in COPD pathogenesis, but should be considered a preventable health risk. Some characteristics and therapeutic alternatives uniquely exist in SHS-related COPD.

## Introduction

Cigarette smoke is the main preventable cause for chronic obstructive pulmonary disease (COPD), resulting in progressive proteolytic, inflammatory, and vasoactive responses that lead to emphysema, small airway obstruction, and pulmonary hypertension. COPD in itself is a serious burden throughout the world, both economically and socially, costing $193 billion in the United States alone (CDC, 2008). The disease is the third largest cause of death in the United States and the fourth worldwide (Pauwels et al., 2001; Minino, 2010). An estimated 95% of COPD cases are attributed to smoking (Barnes et al., 2003), while only a relatively small margin of smokers is susceptible (Fletcher and Peto, 1977). The United States Centers for Disease Control and Prevention considers tobacco smoke to be “the single most important preventable risk to human health in developed countries and an important cause of premature death worldwide,” not only to smokers, but also to those involuntary exposed to second hand smoke (SHS or environmental tobacco smoke, known as ETS) (Oberg et al., 2011).

There are three kinds of smoke that humans are exposed to: mainstream or first hand smoke that is directly inhaled through a person's mouth after taking a puff on a lit cigarette; Side stream smoke, which goes into the air directly from a burning *cigarette, cigar*, or *smoking pipe*; and SHS, which is a combination of both, side stream smoke being the main component of SHS, also known as ETS. Cigarette smoke thereby not only affects smokers, but also contributes to health problems in non-smokers. While a smoker voluntarily inhales the first hand smoke, the non-smoker is inadvertently exposed to SHS that comes from the burning end of a cigarette and the smoke exhaled by the smoker. SHS is therefore also a potential risk factor for COPD and entails symptomatic disease in individuals who are not actually smokers. At present, the dose-response relationship between SHS exposure and COPD is not well established and there is limited understanding of the mechanisms responsible for its pathogenesis, thus halting the development of new advanced treatments for this detrimental disease.

This review is an attempt to sort out the cigarette smoke exposure studies according to levels (low/high) and manner (mainstream or whole body) of the exposure, focusing largely on newer studies applying the rodent model.

## Second hand cigarette smoke exposure

While the qualitative composition of the components is nearly identical in mainstream smoke, side stream smoke, and SHS, the quantitative composition of each is different. In the enclosed environment, due to relatively low ventilation rates (Lofroth, 1989; Jinot and Bayard, 1994), some compounds are emitted at levels up to more than 10 times greater in side stream smoke and SHS when compared with mainstream smoke (Moritsugu, 2007). Side stream smoking has therefore been classified as a Class A *carcinogen* by the *US Environmental Protection Agency*. Still, data regarding the biological evidence linking SHS exposure and COPD are scarce. A 2006 US Surgeon General Report on the health consequences of SHS concluded that the evidence linking SHS and COPD is suggestive (www.surgeongeneral.gov/library/secondhandsmoke), but not sufficient, to infer a causal relationship. The conclusion of the report was drawn primarily from dated epidemiologic evidence that did not establish a biological link (Hirayama, 1981; Kalandidi et al., 1987; Sandler et al., 1989; Robbins et al., 1993; Dayal et al., 1994; Leuenberger et al., 1994; Piitulainen et al., 1998; Berglund et al., 1999). A very recent retrospective analysis of data from 192 countries was published in 2011 and shows that worldwide 40% of children, 33% of male non-smokers, and 35% of female non-smokers are exposed to SHS (Oberg et al., 2011). In 2004 alone, 603,000 deaths were attributable to SHS, accounting for approximately 1% of worldwide mortality. For this reason, the US Surgeon General Report's conclusion should be challenged by providing evidence that SHS exposure can in fact cause COPD by utilizing and comparing animal models as well as assessing patient exposure to SHS.

## The animal model and exposure system

In order to gain insight into the mechanisms of disease development during cigarette smoke exposure, both mainstream and second hand, animal models have been of exceptional use. Animals are exposed to cigarette smoke in a smoking apparatus for either mainstream (nose or head only) or side stream (whole body) applications. In addition, smoke can either be filtered or not, which either depends on the cigarette used (with a filter or without) or the set-up of the apparatus. Another diversity factor is the selection of different animal species with varying susceptibilities, which can be particular to each strain within a species. Early studies of SHS used self-constructed chambers for laboratory animals, allowing environmental smoke to diffuse within a confined space in which the animals were kept. These chambers, unique to each group, did not have the capabilities to measure the parameters essential for assessing cigarette smoke dose and composition. Also, the number and type of cigarettes as well as the length of exposure varied in each study as much as they do today, adding to the difficulty of comparing results. Generally, standardized research-grade cigarettes should be used to easily define a specified dose of total suspended particles (TSP) or total particulate matter (TPM), including nicotine and carbon monoxide levels. Standardized cigarettes became available for worldwide use in 1969 (Roemer et al., 2012) and are most commonly from the University of Kentucky (http://www.ca.uky.edu/refcig/), although there are still often publications using non-reference cigarettes. The introduction of the Teague chamber in 1994 (Teague et al., 1994) has revolutionized the field by allowing maintenance of consistent levels of TSP/TPM that can be set at a variety of concentrations for the exposure of animals to SHS that can mimic human exposures.

In human exposure studies, only the distribution of fine particles (PM_2.5_, which are under 2.5 μm in size and are able to reach the alveoli of the lung) is measured. This accounts for only a small fraction (about 0.1%) of the TSP measurements that are usually reported in animal studies. Data from 66 US casinos with smoking in California, Delaware, Nevada, New Jersey, and Pennsylvania, developed PM_2.5_ frequency distributions, comparing them with three non-smoking casinos. Geometric means for PM_2.5_ were 53.8 μg/m^3^ (range 18.5–205 μg/m^3^) inside smoking casinos, 4.3 μg/m^3^ (range 0.26–29.7 μg/m^3^) outside those casinos, and 3.1 μg/m^3^ (range 0.6–9 μg/m^3^) inside the three non-smoking casinos (Jiang et al., 2010; Lu et al., 2011; Repace et al., 2011; Cochran et al., 2012).

Most commonly for SHS studies listed in Table 2, the Teague chamber was set to exposures between 70 and 150 mg/m^3^. Concurrently, the mainstream smoke exposures listed in Table 1 were as low as 75 mg/m^3^ and as high as 600 mg/m^3^ TSP/TPM (Hodge-Bell et al., 2007). Most of the mainstream smoke studies were performed at 140 mg/m^3^ TSP/TPM levels (Table 1). Ideally, more recent protocols should thereby produce results that can compare the assessed proteolytic, inflammatory, and vasoactive reaction based on similar exposure methods, duration, and cigarette content. This can only be achieved if researchers pertain to the standard procedures available today, such as the use of reference cigarettes in a chamber with defined settings for exposure. These conformities are essential, since a majority of studies are performed in rodents (mice, rats, and guinea pigs), where there is an overwhelming assortment of strains with varying susceptibility, especially when considering mice.

**Table 1:** **Mainstream cigarette smoke exposure modeled in animals**.

**Species/strain**	**Cigarette type**	**Dose and exposure**	**TSP/TPM (mg/m**^**3**^**)**	**Nicotine (mg/m**^**3**^**)**	**CO (ppm)**	**Copd/emphysema**	**Remarks**	**References**
Guinea pig	**X**	2 × 20 ml puffs/min, 8–9 min/cig, 10 min rest, 10/day; 1–60 days	**X**	**X**	**X**			Simani et al., 1974
Sprague-Dawley rat	**X**	12 s puffs, 4 s rest, 2/day, 5 day/week; 25 days	**X**	1.5 mg/cig	**X**		Mainstream presumed	Pittilo et al., 1982
Wistar rat nicorandil p.o.	**Seven star** unfiltered	30 cig, 2 s puff, 15 puffs/min, 8 min	**X**	1.88%	**X**		Mainstream presumed	Gomita et al., 1990
Rat	**X**	8 cig	**X**	1.5 mg/cig	**X**		“Whole smoke”; mainstream presumed; exposure as in Pittilo et al. (1982)	Pittilo et al., 1990
Guinea pig	Commer-cial unfiltered	10/day, 5 day/week; 1–12 month	**X**	**X**	**X**	Emphysema (age and exposure dependent)	mainstream presumed; exposure as in Simani et al. (1974)	Wright and Churg, 1990
Wistar rat	**Long peace**	2 × 20 min, 15 puffs/min; 21 days	**X**	2 mg/cig	**X**		Hamburg II; exposure as in Gomita et al. (1990)	Suemaru et al., 1992
Sprague-dawley rat	Commercial unfiltered	7/day; 1–7 days	**X**	**X**	**X**	PH	“Whole smoke”; exposure as in Simani et al. (1974)	Sekhon et al., 1994
Guinea pig	**X**	10/day, 5 day/week; 4–8 months	**X**	**X**	5% CHG	Emphysema and arteriole muscularization	Exposure as in Wright and Churg (1990)	Wright and Sun, 1994
C57Bl/129 MMP-12 KO with i.t. MCP-1	**KY** unfiltered	2/day, 6 days/week; 6 months	**X**	**X**	10–14% CHG	100% protected	Mainstream presumed; exposure as in Wright and Churg (1990)	Hautamaki et al., 1997
C57Bl/129 MMP-12 KO	**KY** unfiltered	2/day, 6 days/week; 6 month	**X**	**X**	10–14% CHG	100% protected	Mainstream presumed; exposure as in Wright and Churg (1990)	Hautamaki et al., 1997
Sprague-Dawley rat	**X**	20 ml/10 min, 7/day, 5 days/week; 2–12 months	**X**	**X**	4% CHG		Exposure as in Wright and Churg (1990)	Wright et al., 1997
Cam hartley guinea pig	Commercial unfiltered	7/day, 5 days/week; 6 months	**X**	**X**	**X**	COPD and PH	Exposure as in Wright and Churg (1990)	Yamato et al., 1997
Sprague-Dawley rat	Unfiltered	10/day, 5 day/week; 1–6 months	**X**	**X**	10.1 ± 1.5% CHG	Emphysema	Exposure as in Simani et al. (1974)	Ofulue et al., 1998
Sprague-Dawley rat PMN Ab	Unfiltered	10/day, 7 days/week; 2 months	**X**	**X**	**X**	Emphysema		Ofulue and Ko, 1999
Sprague-Dawley rat MoMac Ab	Unfiltered	10/day, 7 day/week; 2 months	**X**	**X**	**X**	Protected		Ofulue and Ko, 1999
Sprague-Dawley rat	**X**	10/day; 24 h	**X**	1.1 mg/cig	11 mg/cig	Small airway constriction	Exposure as in Wright et al. (1997)	Wright et al., 1999
Guinea pig	**Canada Tobacco** unfiltered	7/day, 5 day/week; 24 h-4 months	**X**	1.1 mg/cig	11 mg/cig	Pulmonary arteriole muscularization/hyperplasia	“Whole smoke”	Wright and Sun, 1999
C57Bl/6 × DBA/2 hexavalent chromium i.p.	Commercial **Arda-Bulgar-tabac** filtered	50 ml/cig, 10 min × 9/day; 5 days	533	1.6 mg/cig	**X**		“Whole body mainstream”	Balansky et al., 2000
C57Bl/6	**KY** 2R1	2/day or 1–3/day; 6–48 h	**X**	**X**	**X**	Emphysema via neutrophil elastase	“Whole smoke”; exposure as in Sekhon et al. (1994)	Dhami et al., 2000
C57Bl/6 PMN Ab	**KY** 2R1	2/day or 1–3/day; 6–48 h	**X**	**X**	**X**	Reduced emphysema	“Whole smoke”; exposure as in Sekhon et al. (1994)	Dhami et al., 2000
C57Bl/6 A1AT i.p.	**KY** 2R1	2/day or 1–3/day; 6–48 h	**X**	**X**	**X**	Protected	“Whole smoke”; exposure as in Sekhon et al. (1994)	Dhami et al., 2000
C57Bl/129 TNFR KO and 129J	**KY** 2R1	4/day; 24 h	**X**	**X**	**X**	Protected	“Whole smoke”; exposure as in Dhami et al. (2000)	Churg et al., 2002a
C57Bl/129 metallo-protease inhibitor RS113456	**KY** 2R1	4/day; 24 h	**X**	**X**	**X**	Protected	“Whole smoke”; exposure as in Sekhon et al. (1994)	Churg et al., 2002b
C57Bl/129 MME Tg	**KY** 2R1	4/day; 24 h	**X**	**X**	**X**	Emphysema	“Whole smoke”; exposure as in Sekhon et al. (1994)	Churg et al., 2002b
C57Bl/129 MME KO	**KY** 2R1	4/day; 24 h	**X**	**X**	**X**	Protected	“Whole smoke”; exposure as in Sekhon et al. (1994)	Churg et al., 2002b
Hartley guinea pig p.o. serine elastase inhibitor ZD0892	**KY** 2R1	20 ml/1.5 min, 5/day, 5 day/week; 1 day- 6 months	**X**	**X**	**X**	45% protected	“Whole smoke”; exposure as in Wright and Sun (1999)	Wright et al., 2002
Hartley guinea pig	**Canada Tobacco** unfiltered	20 puff/cig, 10/day, 5 day/week; 4–8 months	**X**	1.1 mg/cig	11 mg/cig	Partial recovery after cessation	Exposure as in Wright and Sun (1994)	Wright and Churg, 2002
C57Bl/129 MMP-12 KO	**KY** 2R1	4 in 1 h; 2–24 h (harvest)	**X**	**X**	**X**	No emphysema	“Whole smoke”	Churg et al., 2003a
C57Bl/6 CD-1 α_1_ antitrypsin (prolastin)	**KY** 2R1	2/day, 5 day/week; 6 months	**X**	**X**	**X**	67% protected	“Whole smoke”; exposure as in Sekhon et al. (1994)	Churg et al., 2003b
C57Bl/6 NE KO	**KY** unfiltered	2/day, 6 day/week; 6 months	**X**	**X**	10% CHG	59% protected	Mainstream presumed; exposure as in Hautamaki et al. (1997)	Shapiro et al., 2003
C57Bl/6 MMP-12 KO	**KY** unfiltered	2/day, 6 day/week; 6 months	**X**	**X**	10% CHG	100% protected	Mainstream presumed; exposure as in Hautamaki et al. (1997)	Shapiro et al., 2003
C57Bl/129 TNFR KO	**KY** 2R1	4/day, 5 day/week; 6 months	**X**	**X**	**X**	71% protected	“Whole smoke”; exposure as in Churg et al. (2002b)	Churg et al., 2004
SHR and Wistar-Kyoto rat	**Long Peace** filtered	23 (5 rat), 26, 30/day (10 rat) 20 min/day, 5 day/week; 8–14 weeks	**X**	1.9 mg/cig	**X**		Hamburg II; exposure as in Suemaru et al. (1992)	Tanaka et al., 2004
Balb/C SCID	**KY** 2R4F unfiltered	5 cig × 4/day, 30 min rest, 5 day/week (1. week 1/day); 5 weeks-6 months	**X**	**X**	8.3 ± 1.4 CHG	Emphysema	Exposure as in D'Hulst et al. (2005b)	D'Hulst et al., 2005a
C57Bl/6	**KY** 1R3	5 cig × 4/day, 30 min rest, 5 day/week; 1 day-24 weeks	**X**	**X**	**X**	Inflammatory cells progressively accumulate	Mainstream presumed (Kobayashi chamber)	D'Hulst et al., 2005b
Sprague-Dawley rat Simvastatin	**Eighty Eight Lights** South Korea	10/day; 16 weeks	**X**	**X**	**X**	100% protected	“Whole smoke”; mainstream presumed; exposure as in Pittilo et al. (1990)	Lee et al., 2005
Balb/C ovalbumin i.p. d0 and d7	**KY** 1R3	5 cig × 4/day, 5 day/week; 10 days	**X**	**X**	**X**	Airway inflammation		Moerloose et al., 2005
C57Bl/6J	**KY** 2R1	2 × 2/day, 10 puffs each, 5 day/week; 2–6 months	**X**	**X**	**X**	Progressive emphysema		van der Strate et al., 2006
Hartley guinea pig MMP-9/-12 inhibitor AZ11557272	**KY** 2R1	7/day, 5 day/week; 1–6 months	**X**	**X**	**X**	68% protected (70% against SAR)	Mainstream presumed; exposure as in Wright and Churg (1990)	Churg et al., 2007a
C57Bl/6 and ICR	**KY** 2R4F	2 h/day, 5 day/week; 6 months	75, 250, 600	**X**	**X**	Mild emphysema		Hodge-Bell et al., 2007
C57Bl/6 IL-18Ra KO	**KY** 2R4 unfiltered	2 × 2/day, 5 day/week; 6 months	**X**	**X**	**X**	51% protected	Mainstream presumed; exposure as in Hautamaki et al. (1997)	Kang et al., 2007
C57Bl/6 CD8 KO	**KY** unfiltered	2/day, 6 day/week; 6 months	**X**	**X**	10% CHG	100% protected	Mainstream presumed; exposure as in Hautamaki et al. (1997)	Maeno et al., 2007
C57Bl/6 CD4 KO	**KY** unfiltered	2/day, 6 day/week; 6 months	**X**	**X**	10% CHG	Emphysema	Mainstream presumed; exposure as in Hautamaki et al. (1997)	Maeno et al., 2007
FVB MrpI/MdrIa/Ib KO	**KY** 2R1	2 cig × 2/day, 10 puffs each, 5 day/week; 6 months	**X**	**X**	**X**	No emphysema or inflammation		van der Deen et al., 2007
C57Bl/6J influenza or viral PAMP	**KY** 2R4 unfiltered	1. week 0.5 cig × 2/day 2. week 1 cig × 3/day	**X**	**X**	**X**	Accelerated emphysema	Mainstream presumed; exposure as in Hautamaki et al. (1997)	Kang et al., 2008
C57Bl/6	**KY** 1R3	2 or 4 cig 5 day/week; 2–6 months	**X**	**X**	**X**	T and B lymphocyte response	Exposure as in Simani et al. (1974); Hautamaki et al. (1997)	Zavitz et al., 2008
C57Bl/6 i.p. caspase inhibitor	**KY** 2R1	4/day acute or 3/day, 5 day/week; 24 h	**X**	**X**	**X**	100% protected	“Whole smoke”; mainstream presumed	Churg et al., 2009a
C57Bl/6 TNFR KO	**KY** 2R1	4/day acute or 3/day, 5d/week; 6 months	**X**	**X**	**X**	83% protected (100% against SAR)	“Whole smoke”; mainstream presumed	Churg et al., 2009b
C57Bl/6 IL-1R KO	**KY** 2R1	4/day acute or 3/day, 5 day/week; 24 h-6 months	**X**	**X**	**X**	65% protected (100% against SAR)	“Whole smoke”; mainstream presumed	Churg et al., 2009b
C57Bl/6	**KY** 2R1	4/day acute or 3/day, 5 day/week; 2 h-6 months	**X**	**X**	**X**	Emphysema	“Whole smoke”; mainstream presumed	Churg et al., 2009b
C57Bl/6 clarithro-mycin p.o.	**KY** unfiltered	2/day, 6 day/week; 6 months	**X**	**X**	**X**	Reduced emphysema	Mainstream presumed; exposure as in Hautamaki et al. (1997); Shapiro et al. (2003)	Nakanishi et al., 2009
C57Bl/6J curcumin p.o.	Commercial filtered **Marlboro**	12 puffs/min, 60 min/day, 10 day or 5 day/week; 10 day-12 weeks	971 ± 98.3 in 5% CS	1 mg/cig 104.5 ± 49.3 ng/ml cotinin	**X**	Reduced emphysema		Suzuki et al., 2009
C57Bl/6 MMP-9 KO	**KY** 3R4F unfiltered	4/day, 6 day/week; 6 months	**X**	**X**	**X**	Emphysema	Exposure as in Hautamaki et al. (1997)	Atkinson et al., 2010
C57Bl/6 adipo-nectin KO	**KY** 2R4F	35 ml puff/25 s, 5 min/cig, 2/day, 5 day/week; 6 months	173 ± 5.3 (100–250)	2.45 mg/cig	**X**	Reduced emphysema and inflammation		Miller et al., 2010
C57Bl/6J SOD3 Tg	**KY** 3R4F	2 × 1 h/day; 3 day-6 months	300 (3 day) or 100 (month)	**X**	**X**	Reduced emphysema		Yao et al., 2010
C57Bl/6J iNOS KO	**KY** 3R4F	6 h/day, 5 day/week; 8 months	140	**X**	**X**	Protected emphysema and PH		Seimetz et al., 2011
C57Bl/6J eNOS KO	**KY** 3R4F	6 h/day, 5 day/week; 8 months	140	**X**	**X**	Emphysema and PH		Seimetz et al., 2011
C57Bl/6J iNOS inhibitor L-NIL	**KY** 3R4F	6 h/day, 5 day/week; 8 months	140	**X**	**X**	Protected emphysema and PH		Seimetz et al., 2011
Hartley guinea pig AZD9668 NE inhibitor p.o.	**KY** R21	2 h/day, 5 day/week; 24 weeks	**X**	**X**	**X**	Protected emphysema and SAR	Exposure as in Churg et al. (2007a)	Stevens et al., 2011
Hartley guinea pig simvastatin	**KY** 2R1 and 2R4F	5/day, 5 day/week; 6 months	**X**	**X**	**X**	Protected PH and emphysema, *not* SAR	Exposure as in Barnes (1990) (review)	Wright et al., 2011
Hartley guinea pig MPO inhibitor AZ1	**KY** 2R1	5/day, 5 day/week; 6 months	**X**	**X**	**X**	Reduced emphysema, SAR, PH	Mainstream presumed; exposure as in Simani et al. (1974)	Churg et al., 2012
Hartley guinea pig	**KY** 1R3F unfiltered	7/day, 5 day/week; 3–6 months	**X**	**X**	**X**	COPD		Dominguez-Fandos et al., 2012
C57Bl/6	**KY** 2R1	4/day; 24 h	**X**	**X**	**X**		Exposure as in Churg et al. (2003b)	Preobrazhenska et al., 2012
C57Bl/6	**Marlboro** 100	4/day 4–5 min, 5 day/week; 5 s puff, 10 min rest; 4 months	**X**	**X**	**X**	Emphysema	”Active smoke“	Shan et al., 2012
C57Bl/6 IL17a KO	**Marlboro** 100	4/day 4–5 min, 5 day/week; 5 s puff, 10 min rest; 4 months	**X**	**X**	**X**	Reduced emphysema	”Active smoke“	Shan et al., 2012
C57Bl/6 IL17a Tg	**Marlboro** 100	4/day 4–5 min, 5 day/week; 5 s puff, 10 min rest; 4 months	**X**	**X**	**X**	Exacerbated emphysema	”Active smoke“	Shan et al., 2012
C57Bl/6 Tcrd KO	**Marlboro** 100	4/day 4–5 min, 5 day/week; 5 s puff, 10 min rest; 4 months	**X**	**X**	**X**	Exacerbated emphysema	”Active smoke“	Shan et al., 2012
C57Bl/6 Spp1 KO	**Marlboro** 100	4/day 4–5 min, 5 day/week; 5 s puff, 10 min rest; 6 months	**X**	**X**	**X**	Reduced emphysema	”Active smoke“	Shan et al., 2012
Mouse eNOS KO	**KY** 2R1 and 2R4F	5/day, 5 day/week; 6 months	**X**	**X**	**X**	Pulmonary hypertension, COPD	Exposure as in Barnes (1990) (review)	Wright et al., 2012
Balb/C	**Marlboro red**	9 cig/day, 10 s CS and 50 s fresh air; 4 days	**X**	1 mg/cig	**X**			Nemmar et al., 2013

CHG, carboxyhemoglobin; PH, pulmonary hypertension; SAR, small airway remodeling. Cigarette brand names are in bold.

Today, rodents are the most commonly used models. While mice are surely favored for their wide variety of applicable gene expression manipulations, it remains difficult to standardize measurements of pulmonary function to assess disease parameters. The guinea pig model is also occasionally applied, mainly by one group of investigators (Simani et al., 1974; Wright and Churg, 1990, 2002; Wright and Sun, 1994, 1999; Wright et al., 2002, 2011) though increased inflammatory cells and muscularization of pulmonary vessels was recently documented (Dominguez-Fandos et al., 2012). The rat is a favorable model, since measurable emphysematous changes which further progress can be detected after only 2 months of smoke exposure (Kratzer et al., 2013).

Mainstream smoke and SHS exposure studies are summarized in Tables 1, 2, respectively. Animal studies with an unidentified smoke exposure are presented in Table 3. The cigarette brands used are listed in Table 4.

**Table 2:** **Second hand cigarette smoke exposure modeled in animals**.

**Species/strain**	**Cigarette type**	**Dose and exposure**	**TSP/TPM (mg/m**^**3**^**)**	**Nicotine (mg/m**^**3**^**)**	**CO (ppm)**	**COPD/emphysema**	**Remarks**	**References**
Wistar rat	Commercial **Virginia**	3/day in 90 min, 5 day/week; 3 months	**X**	1 g/cig	35	Emphysema	Nicotine levels correct?	Escolar et al., 1995
A/J	**KY** 1R4F	6 h/day, 5 day/week; 6 months	4.1 ± 0.4	1.011 ± 0.289	17 ± 2			Witschi et al., 1995
Guinea pig	Commercial unfiltered	20/day, 5 day/week; 1–8 weeks	**X**	**X**	16.3 ± 4.9% CHG	Emphysema	“Whole smoke”	Selman et al., 1996
A/J	**KY** 1R4F	6 h/day, 5 day/week; 5 month (harvest 9 months)	78.5 ± 12.4	13.4 ± 3.3	211 ± 24		“Whole smoke”; exposure as in Witschi et al. (1995)	Witschi et al., 1997
A/J	**KY** 1R4F	6 h/day, 5 day/week; 5 month (harvest 9 months)	0.1 ± 0.2	3.1 ± 2	113 ± 23		”Filtered smoke”	Witschi et al., 1997
Sprague-Dawley rat hexavalent chromium i.t.	**KY** 2R1 humidified	10 cig × 2/day, 3 h rest, 23 mm butt; 18 days	120	2.45 mg/cig	**X**		”Whole body environmental”	Balansky et al., 2000
C57Bl/6, DBA/2, ICR	Commercial **Virginia**	33 ml/min, 3/day, 5 day/week; 7 months		0.9 mg/cig	**X**	ICR resistant to emphysema (anti-oxidants)	“Macrolon cages”; exposure as in Escolar et al. (1995)	Cavarra et al., 2001
Pallid mouse α_1_ proteinase inhibtor deficiency	Commercial **Virginia**	33 ml/min, 3/day, 5 day/week; 4 months		0.9 mg/cig	**X**	More severe emphysema	“Macrolon cages”; exposure as in Escolar et al. (1995)	Cavarra et al., 2001
NZ White rabbit	**Ultratech Corp** research cigarettes	6 h/day, 48/day, 5 day/week; 10 weeks	24.08 ± 3.79	339 ± 74.6 nmol/L plasma	44.91 ± 1.81			Sun et al., 2001
SH rat catalytic anti-oxidant i.t. (AEOL 10150)	**KY** 1R4F humidified	35 ml 2 s puff/min, 6 h/day, 3 day/week; 2 day-8 weeks	68.6 ± 11.7	5.7 ± 2.0	275 ± 39	Reduced lung injury		Smith et al., 2002
Balb/C a-tocopherol	Commercial filtered	5/day over 60 min; 10 weeks	**X**	**X**	**X**			Koul et al., 2003
ICR Nrf2 KO	**KY** 2R4F	35 m^3^ 2 s puff × 8, 1 min rest, 7 h/day, 7 day/week; 6 months	90	2.45 mg/cig	350	More severe emphysema	Teague chamber; exposure as in Witschi et al. (1997)	Rangasamy et al., 2004
C57Bl/6J	Commercial **Virginia** filtered	3/day, 5 day/week; 1–10 months	**X**	0.9 mg/cig	**X**	Disseminated emphysema	“Macrolon cages”; exposure as in Cavarra et al. (2001)	Bartalesi et al., 2005
DBA/2	Commercial **Virginia** filtered	3/day, 5 day/week; 1–10 months	**X**	0.9 mg/cig	**X**	Uniform emphysema	“Macrolon cages”; exposure as in Cavarra et al. (2001)	Bartalesi et al., 2005
C57Bl/CBA and A/J	**X**	6 h/day, 5 day/week; C57Bl/CBA 12 month; A/J 6 months	250	**X**	10–12% CHG	Emphysema (A/J w/o SAR)	Teague chamber	Foronjy et al., 2005
B6C3F and A/J	**KY** 1R3, unfiltered	6 h/day, 5 day/week; 15 weeks	1. week 100–125, then 250	**X**	**X**	A/J 51% greater mean linear intercept, B6C3F 38%		March et al., 2005
B6C3F all trans-retinoic acid inhalation	**KY** 1R3, unfiltered	6 h/day, 5 day/week; 32 weeks	1. week 100–125, then 250	**X**	**X**	No emphysema reversal		March et al., 2005
A/J all trans-retinoic acid i.p. and inhalation	**KY** 1R3, unfiltered	6 h/day, 5 day/week; 15 weeks	1. week 100–125, then 250	**X**	**X**	No emphysema reversal		March et al., 2005
C57Bl/6 p.o. Rofluminlast (PDE4 inhibitor)	Commercial **Virginia** filtered	5 in 20 min or 3/day, 5 day/week; 4 h-7 months	**X**	0.9 mg/cig	**X**	100% protected	“Macrolon cages”; side stream presumed; exposure as in Cavarra et al. (2001)	Martorana et al., 2005
SH rat sEH inhibitor s.c.	**KY** 2R4F humidified	35 ml 2 s puff/min, 6 h/day; 3 days	76.4 ± 16.0	6.8 ± 0.2	234 ± 2	Attenuated inflammation		Smith et al., 2005
Sprague-Dawley rat CXCR2 antagonist SB-332235	**KY** 2R4F filtered	2–5 cig/day, 50 ml/30 s over 32 min; 24 h 3–4 days (unclear)	**X**	**X**	**X**	Reduced COPD (time and dose dependent)	No mention of number of cigarettes; exposure period unclear	Stevenson et al., 2005
C57Bl/6 CCR6 KO	**KY** 2R4F unfiltered	5 cig × 4/day, 30 min rest, 5 day/week; 4 week-6 months	**X**	**X**	8.3 ± 1.4 CHG	67% protected (none against SAR)	Exposure as in D'Hulst et al. (2005b)	Bracke et al., 2006
C57Bl/6 TNFR KO	**KY** 2R4F unfiltered	5 cig × 4/day, 30 min rest, 5 day/week; 3–6 months	**X**	**X**	8.3 ± 1.4 CHG	100% proteced	Side stream presumed; exposure as in D'Hulst et al. (2005b)	D'Hulst et al., 2006
C57Bl/CBA SOD Tg	**KY** 2R4F	2 × 70 ml puffs/min, 6 h/day, 5 day/week; 12 months	250	**X**	10% CHG	Prevents emphysema	Teague chamber	Foronjy et al., 2006
A/J	**KY** 1R3 unfiltered	10–22 week (+39 weeks harvest)	1. week 100, then 250	**X**	**X**	Emphysema (concentration and duration dependent)	“Whole body mainstream”	March et al., 2006
A/J female	**KY** 1R3 unfiltered	10–22 weeks	1. week 100, then 250	**X**	**X**	Emphysema less severe (concentration and duration dependent)	“Whole body mainstream”	March et al., 2006
A/J female p.o. EGCG anti-oxidant	**KY** 1R3 unfiltered	16 weeks	1. week 100, then 250	**X**	**X**	Emphysema (concentration and duration dependent)	“Whole body mainstream”	March et al., 2006
A/J female NAC anti-oxidant p.o.	**KY** 2R4F filtered	10 weeks	1. week 100, then 250	**X**	**X**	Emphysema (concentration and duration dependent)	“Whole body mainstream”	March et al., 2006
A/J female	**KY** 2R4F filtered	10 weeks	1. week 100, then 250	**X**	**X**	Emphysema (concentration and duration dependent)	“Whole body mainstream”	March et al., 2006
C57Bl/6 CCR5 KO	**KY** 2R4F unfiltered	5 cig × 4/day, 30 min rest, 5 day/week; 4 week-6 months	**X**	**X**	8.3 ± 1.4 CHG	25% protected (none against SAR)	Exposure as in D'Hulst et al. (2005b)	Bracke et al., 2007
C57Bl/6, A/J, AKR, CD-1 (ICR), 129Sv	**KY** 2R4F	35 ml 2 s puff/min; 3 days (2 and 24 h harvest)	80: 6 h/day or 300: 2 × 1 h/day	0.85 mg/cig	297 (300 TPM); 79.4 (80 TPM)	C57Bl/6 highly susceptible to inflammatory and oxidative response; A/J, AKR, CD-1 (ICR) less susceptible; 129Sv resistant		Yao et al., 2008
C57Bl/6, Balb/C, A/J, 129Sv	**KY** 1R3F	40 ml puffs/min; 2, 3, 4, or 5 cig/day 1 h/day; 3 days	481	**X**	**X**	Neutrophilia dose- and time-dependent	Exposure as in Stevenson et al. (2005)	Morris et al., 2008
C57Bl/6 and Balb/C PKF242-484 MMP inhibitor p.o. and i.n.	**KY** 1R3F	40 ml puffs/min; 2, 3, 4, or 5 cig/day 1 h/day; 3 days	481	**X**	**X**	Strain-dependent inhibition of neutrophil inflammation	Exposure as in Stevenson et al. (2005)	Morris et al., 2008
Hartley guinea pig	**KY** 2R4F	2 × 70 ml puff/min, 4 h/day, 5 day/week; 1–12 weeks	250	**X**	**X**	Inflammation (4 week), emphysema (12 week)	Teague chamber	Golovatch et al., 2009
Balb/C	**KY** 1R4F	6 h/day, 5 day/week; 12 weeks	30 ± 1	4.8 ± 0.5	**X**	CS augments inflammatory cell recruitment in COPD		Rao et al., 2009
C57Bl/6J 10^6^ apoptotic thymocytes i.t.	**KY** 3R4F	5 h; 1 day (harvest and i.t. d0–5)	25 or 100	**X**	**X**	Reversible and cell type independent impaired efferocytosis in COPD	Teague chamber	Richens et al., 2009
C57Bl/6J 10^6^ apoptotic thymocytes i.t.	**KY** 3R4F	5 h/day; 5 day (harvest +1 and 4 weeks)	100	**X**	**X**	Reversible impaired efferocytosis in COPD	Teague chamber	Richens et al., 2009
FVB/N apoptotic neutrophils	**KY** 3R4F	5 h/day, 5 day/week; 22 week (harvest + 20 weeks)	1. week 100, then 250	**X**	**X**	Non-reversible impaired efferocytosis and termnal bronchiolitis in COPD	Teague chamber	Richens et al., 2009
ICR oxidant resistant	**KY** 3R4F	5 h; 1 day (harvest d0–2)	100	**X**	**X**	No COPD	Teague chamber	Richens et al., 2009
C57Bl/6 i.p. MnTBAP	**KY** 3R4F	5 h; 1 day (harvest d0–2)	100	**X**	**X**	Anti-oxidant treatment clears apoptotic cells and inhibits RhoA	Teague chamber	Richens et al., 2009
ecSOD Tg	**KY** 3R4F	5 h; 1 day (harvest d0–2)	100	**X**	**X**	Anti-oxidant treatment clears apoptotic cells and inhibits RhoA	Teague chamber	Richens et al., 2009
C57Bl/129 TNFR KO	**KY** 3R4F	5 h; 1 day (harvest d0–2)	100	**X**	**X**	CS inhibition of efferocytosis is TNF-α dependent	Teague chamber	Richens et al., 2009
C57Bl/6J	**KY** 2R4F humidified	35 ml 2 s puff/min, 8 puff/cig, 6 h/day; 3 months	Med 69; high 131	2.5–6.8 mg/cig	Med 238; high 394	Inflammatory COPD	Teague chamber	Woodruff et al., 2009
A/J	**KY** 2R4F	1. week 125 4 h/day, 5 day/week; 20 week (harvest 20 or 28 weeks)	750	40 μg/l	800	Smoking cessation stops emphysema progression and reduces inflammation	Performed with maintream and side stream	Braber et al., 2010
Balb/C and C57Bl/6	**KY** 2R4F filtered	12 cig × 2/day 1. day 20 min 2. day 30 min, then 50 min, 5 day/week; 4 day-24 weeks	622 ± 90	377–503.2 ng/ml cotinin	10–15% CHG	Inflammation adaptive (T regulatory lymphocytes)		Botelho et al., 2010
C57Bl/6 P2Y_2_R KO	Commercial **Virginia** filtered	3/day, 5 day/week; 3 day-7 months	**X**	0.9 mg/cig	**X**	Protected	“Whole smoke”; side stream presumed; exposure as in Cavarra et al. (2001)	Cicko et al., 2010
Sprague-Dawley rat	**KY** 2R4F	4/day, 5 day/week; 3 day-6 months	27.1 ± 0.8/cig	2.66 ± 0.12 μM cotinin	42 ± 4 μM CHG	Emphysema w/o apoptosis		Marwick et al., 2010
Balb/C Rag KO with CS CD3+ T lymphocyte	**KY** 3R4F	4 h/day, 5 day/week; 6 month (+13 week post T cell transfer)	150 ± 15	**X**	400 ± 30	Emphysema	Teague chamber	Motz et al., 2010a
C57Bl/6J	**KY** 3R4F	4 h/day, 5 day/week; 2–24 weeks	150 ± 15	**X**	**X**	NK cells activate innate immune system in COPD	Teague chamber	Motz et al., 2010b
Sprague-Dawley rat celecoxib i.g.	Commercial **Eighty Eight Lights**	10/day, 2 h/day, 5 day/week; 20 weeks	**X**	**X**	**X**	Reduced emphysema		Roh et al., 2010
C57Bl/6J SOD3 Tg	**KY** 3R4F	5 h/day, 5 day/week; 2–6 months	100	**X**	**X**	Protected	Teague chamber	Yao et al., 2010
C57Bl/129 Rtp801 KO	**KY** 2R4F unfiltered	4/day, 5 h/day; 6 months	**X**	2.45 mg/cig	**X**	Protected	Teague chamber; exposure as in Rangasamy et al. (2004)	Yoshida et al., 2010
A/J	**KY** 2R4F, whole body	4 h/day, 5 day/week; 20 week (harvest 20 or 28 weeks)	2. week 125, then 750	40 μg/l	800	Emphysema	“Whole body mainstream”	Braber et al., 2011
C57Bl/6 IL-1R1 KO	**KY** 2R4F unfiltered	12 cig × 2/day 50 min, 5 day/week; 4 day-8 weeks	622 ± 90	377–503.2 ng/ml cotinin	10–15% CHG	IL-1R1/IL-1α dependent inflammation in COPD	Exposure as in Botelho et al. (2010)	Botelho et al., 2011a
C57Bl/6 IL-1α KO; Balb/C IL-1α Ab i.p.	**KY** 2R4F unfiltered	12 cig × 2/day 50 min, 5 day/week; 4 day-8 weeks	622 ± 90	377–503.2 ng/ml cotinin	10–15% CHG	IL-1R1/IL-1α dependent inflammation in COPD	Exposure as in Botelho et al. (2010)	Botelho et al., 2011a
C57Bl/6 IL-1β KO; Balb/C IL-1β Ab i.p.	**KY** 2R4F unfiltered	12 cig × 2/day 50 min, 5 day/week; 4 day-8 weeks	622 ± 90	377–503.2 ng/ml cotinin	10–15% CHG	IL-1β independent inflammation in COPD	Exposure as in Botelho et al. (2010)	Botelho et al., 2011a
Balb/C GM-CSF and GM-CSFR Ab i.p.	**KY** 2R4F unfiltered	12 cig × 2/day 1. day 20 min 2. day 30 min then 50 min; 4 days	622 ± 90	377–503.2 ng/ml cotinin	10–15% CHG	Reduced inflammatory response	Exposure as in Botelho et al. (2010)	Botelho et al., 2011b
DBA/2J hyaluronan	**KY** 2R4F filtered	35 ml 2 s puff/min, 3 h/day, 5 day/week; 2–10 months	**X**	**X**	**X**	Reduced emphysema	Teague chamber	Cantor et al., 2011
DBA/2 caspase-3 inhibition	**KY**	33 ml/min, 3/day, 5 day/week; 6 months	90	**X**	350	Reduced emphysema	Teague chamber; exposure as in Cavarra et al. (2001)	Clauss et al., 2011
SH rat sEH inhibitor s.c. or p.o.	**KY** 3R4F	6 h/day; 3 days	80–90	**X**	**X**	sEH anti-inflammatory effect independent of leukocyte recruitment	Teague chamber	Davis et al., 2011
C57Bl/6 P2X7 receptor KO	**KY** 3R4F unfiltered	250, 500, 750 ml/min, 50 min × 2/day; 3 days	**X**	**X**	**X**	Inflammation through P2X7 pathway	Teague chamber	Eltom et al., 2011
C57Bl/129 Smad3 KO	**KY** 2R4F unfiltered	12 cig × 2/day 50 min, 5 day/week; 4 day-8 weeks	622 ± 90	377–503.2 ng/ml cotinin	10–15% CHG	Accelerated emphysema	Exposure as in Botelho et al. (2010)	Farkas et al., 2011
C57Bl/6 and DR4 Tg M. tuberculosis or influenza A i.n.	**KY** 1R4F	2 × 120 min/day (2 h rest) 5 day/week; 6 weeks	80	**X**	**X**		Teague chamber	Feng et al., 2011
C57Bl/CBA	**KY** 2R4F	6 h/day, 5 day/week; 4 week-12 months	**X**	**X**	**X**		Teague chamber; exposure as in Foronjy et al. (2005, 2006), Golovatch et al. (2009)	Geraghty et al., 2011
Hartley Guinea pig	**KY** 2R4F	6 h/day, 5 day/week; 12 weeks	**X**	**X**	**X**		Teague chamber; exposure as in Foronjy et al. (2005, 2006); Golovatch et al. (2009)	Geraghty et al., 2011
C57Bl/6 and DBA/2J adipose stem cell treatment	**KY** 3R4F	4, 6 months	90	**X**	350	Reduced emphysema	Teague chamber	Schweitzer et al., 2011
Balb/C AZD9668 NE inhibitor p.o.	**KY** 1R3F	12 cig 2 × 50 min/day; 4 days	**X**	**X**	**X**	Reduced emphysema and SAR		Stevens et al., 2011
C57Bl/6 IL-1R1 and IL-1α KO	**KY** 3R4F unfiltered	12 cig × 2/day 50 min, 5 day/week; 4 day-8 weeks	622 ± 90	377–503.2 ng/ml cotinin	10–15% CHG	DC accumulation and activation is IL-1R1/IL-1α dependent	Exposure as in Botelho et al. (2010)	Botelho et al., 2012
C57Bl/6 TLR4 KO	**KY** 3R4F unfiltered	12 cig × 2/day 50 min, 5 day/week; 4 day-8 weeks	622 ± 90 μg/l TPM	377–503.2 ng/ml cotinin	10–15% CHG	DC accumulation and activation is TLR4-independent	Exposure as in Botelho et al. (2010)	Botelho et al., 2012
Balb/C IL-1α Ab i.p.	**KY** 3R4F unfiltered	12 cig × 2/day 50 min; 4 days	622 ± 90 μg/l TPM	377–503.2 ng/ml cotinin	10–15% CHG	DC accumulation and activation is IL-1α dependent	Exposure as in Botelho et al. (2010)	Botelho et al., 2012
Balb/C IL-1β Ab i.p.	**KY** 3R4F unfiltered	12 cig × 2/day 50 min; 4 days	622 ± 90 μg/l TPM	377–503.2 ng/ml cotinin	10–15% CHG	DC accumulation and activation is IL-1β-independent	Exposure as in Botelho et al. (2010)	Botelho et al., 2012
SH rat	**KY** 3R4F	35 ml 2 s puff, 6 h/day, 3 day/week; 3 day-12 weeks	80–90	**X**	**X**	Leukocytes from bronchial circulation in COPD	Nicotine and CO levels measured daily, but not listed; Teague chamber	Davis et al., 2012
Sprague-Dawley rat	**Ye Shu** unfiltered	1. day 3/h each; 2. day: 7/h each; 3–5. day: 12/3 min; 6. day-end: 12/h; 4–6 weeks	70–110	1.2 mg/cig	310–380	COPD	Chamber uniquely described; side stream presumed	Nie et al., 2012
SH rat sEH inhibitor and Rolipram p.o.	**KY** 3R4F	35 ml 2 s puff/min, 6 h/day, 3 day/week; 4 weeks	80–90	**X**	**X**	Reduced emphysema	Teague chamber	Wang et al., 2012
Mouse Apo-E KO C. pneumoniae	**KY** 3R4F	35 ml 2 s puff/min, 6 h/day, 5 day/week; 8 weeks	**X**	**X**	**X**	Enhanced artherosclerosis		Zhao et al., 2012
Balb/C and C57Bl/6	**KY** 3R4F	4 h/day, 5 day/week; 6 months	150 ± 15	**X**	400 ± 30	Emphysema CD4+ and CD8+ T lymphocyte dependent (Ag-specific response)	Teague chamber	Eppert et al., 2013
Sprague-Dawley rat	**KY** 1R3F	5 cig/9 min 6 h/day; 2–4 months	100–120 μg/m^3^	**X**	**X**	Emphysema	Teague chamber	Kratzer et al., 2013
C57Bl/6 IKK-2 KO	**KY** 3R4F unfiltered	500 ml/min, 50 min; 3–14 days	**X**	**X**	**X**	Unaltered inflammation	Teague chamber;exposure as in Eltom et al. (2011)	Rastrick et al., 2013

CHG, carboxyhemoglobin; SAR, small airway remodeling. Cigarette brand names are in bold.

**Table 3:** **Animal studies where the exposure (mainstream or second hand smoke) is not known**.

**Species/strain**	**Cigarette type**	**Dose and exposure**	**TSP/TPM (mg/m**^**3**^**)**	**Nicotine (mg/m**^**3**^**)**	**CO (ppm)**	**COPD/Emphysema**	**Remarks**	**References**
C57Bl/6J A1AT low pallid mouse	**KY** 2R1 unfiltered	2–6 months	**X**	**X**	10–12% CHG	Emphysema	No exposure method	Takubo et al., 2002
C57Bl/6, NZW, A/J, SJ/L, AKR/J	**KY** 2R1 unfiltered	2/day, 5 day/week; 6 months	**X**	**X**	10–12% CHG	Strain-dependent susceptibility to emphysema	No exposure method	Guerassimov et al., 2004
C57Bl/6 and Balb/C INF-g Tg	**X**	6 months	**X**	**X**	**X**	Enhanced emphysema	No exposure method	Ma et al., 2005
C57Bl/6 CCR5 KO	**X**	6 months	**X**	**X**	**X**	100% protected	No exposure method	Ma et al., 2005
C57Bl/6	**KY** 2R1	4/day once or 3/day, 5 day/week; 2 h-6 months	**X**	**X**	**X**	SAR	No exposure method	Churg et al., 2006
C57Bl/6 A1AT i.p.	**KY** 2R1	4 in 2 h	**X**	**X**	**X**	No emphysema	No exposure method	Churg et al., 2007b
C57Bl/6	**KY** 2R1	20 ml × 2 cig/day 15 min, 6 day/week; 6 months	**X**	**X**	**X**	Emphysema	No exposure method	Adair-Kirk et al., 2008

CHG, carboxyhemoglobin; SAR, small airway remodeling. Cigarette brand names are in bold.

**Table 4:** **Cigarette types used for the summarized experiments**.

**Cigarette brand**	**Type**	**Year**	**TSP/TPM (mg/cig)**	**Nicotine (mg/cig)**	**CO (mg/cig)**	**References**
Kentucky	1R3	1974	27.1 and 23.6	1.46 and 1.23	17.1 and 14.7	Davis et al., 1983
Kentucky	1R3F	1974	18.1	1.16	17.2	Davis et al., 1983
Kentucky	1R4F	1983	10.3^*^	0.8^*^	11.6^*^	Davis et al., 1983
Kentucky	2R1	1974	14.6 and 38.8	2.45 and 2.19	25.1 and 22.2	Davis et al., 1983
Kentucky	2R1F	1974	28.6	1.74	22	Davis et al., 1983
Kentucky	2R4F	2001	11.7	0.9	13	Roemer et al., 2012
Kentucky	3R4F	2008	11	0.7	12	Roemer et al., 2012
Virginia	F			0.9	35 (Escolar et al., 1995)	Escolar et al., 1995; Cavarra et al., 2001; Bartalesi et al., 2005; Martorana et al., 2005; Cicko et al., 2010
Seven Star	UF			1.88%		Gomita et al., 1990
Long Peace	F			1.9–2		Suemaru et al., 1992; Tanaka et al., 2004
Ultratech Corp	n.a.		24.08 ± 3.79	339 ± 74.6 nmol/L	44.91 ± 1.81	Sun et al., 2001
Ye Shu	UF		70–110	1.2	310–380	Nie et al., 2012
Marlboro	100F		971 ± 98.3 in 5%	1	12	Suzuki et al., 2009; Shan et al., 2012
Eighty Eight Lights	n.a.			0.72	9	Lee et al., 2005; Roh et al., 2010
Arda-Bulgartabac	F		533	1.6 mg/cig		Balansky et al., 2000
Canada Tobacco	UF			1.1	11	Wright and Churg, 1990

F, filtered (KY 30 mm butt); UF, unfiltered; KY values, 23 and 30 mm butt;

* , 35 mm butt.

## Chronic cigarette smoke exposure

It is important to differentiate acute versus chronic smoke exposure in respect to lung structure and function (Martin and Tamaoka, 2006). Though both models induce airway narrowing to a certain extent, the inflammation seen initially diminishes during the time-course of exposure, along the lines of early repair and late-stage failure to repair (Churg and Wright, 2009; Kratzer et al., 2013). Mechanisms of repair seem to be sufficient for the first month of cigarette smoke exposure, but since lesions change over the exposure time, it is predicted that the pathological mechanisms differ in acute and chronic cases. Acute models can be as short as 24 h or last up to as many as 2 weeks, while chronic models should be standardized to 6 months (24 weeks) of exposure to induce the anatomical changes characterizing COPD (Churg and Wright, 2009). Both acute and chronic cigarette smoke exposure lead to oxidant stress, which pathologically affects airway cells to promote remodeling (Martin and Tamaoka, 2006).

Since COPD can only manifest the pathological characteristics seen in humans after long-term cigarette smoke exposure, animals are often utilized in chronic studies to relate to the human disease. Emphysema, airway remodeling and pulmonary hypertension, which progress over time, can only be induced in chronic models where structural alterations occur. Irreversible matrix destruction, fibrosis, airway wall thickening, and hyperplasia of smooth muscle and goblet cells as well as fibroblasts can only be seen in chronic cigarette exposure. Since COPD in smoking patients is typically GOLD (Global Initiative for Chronic Obstructive Lung Disease) stage III or IV and less commonly I and II (Retamales et al., 2001), animal models must attempt to mimic this by developing emphysema, small airway remodeling (SAR), or pulmonary hypertension. The excess mucus production that defines chronic bronchitis is also thought to be important in the pathogenesis of acute exacerbations of COPD (Burge and Wedzicha, 2003). This was previously considered difficult to reproduce in animal models due to the fact that, in contrast to humans, the anatomical localization of bronchial glands in rats and mice are concentrated in the proximal trachea (Churg et al., 2008). Recently, the mucus secretion was demonstrated in two rat models (Nie et al., 2012). Authors showed significantly increased mucus production in epithelium of trachea, bronchi, and bronchiole in a 6 week cigarette smoke model and in a chronic bronchitis model with 6 weeks cigarette smoke exposure combined with a single intratracheal injection of LPS on day 39. There was also a significant increase in MUC5A protein levels in broncheoalveolar lavage in both models (Nie et al., 2012).

## The anatomical manifestations of COPD

For chronic cigarette smoke exposure studies, many months are required to model the lung pathology, as acute models do not have this effect on lung structure and therefore do not allow the prediction of chronic outcomes. In order to evaluate, for example, drug efficacies, a chronic exposure most reliably modeling the human disease is essential. Only chronic models can present the lesions of COPD defined as emphysema, SAR, and pulmonary hypertension, though still more mildly than in human COPD (Wright and Churg, 2008; Churg and Wright, 2009). This is especially important when noting that, because rodents develop a mild form of COPD, drug tests should be performed as late as possible to mimic a treatment versus a prophylaxis (Churg et al., 2011).

## Emphysema

The pathogenesis of emphysema is studied by inducing the disease with various methods not necessarily restricted to cigarette smoke. The protease/anti-protease hypothesis has been the main focus in elucidating the cause of emphysema. In addition, oxidative stress has been shown to cause lung cell apoptosis in an emphysema model using a vascular endothelial growth factor (VEGF) receptor blocker (Kasahara et al., 2000; Tuder et al., 2003). Cigarette smoke induces both oxidative stress and the infiltration of inflammatory cells, which release proteases that overwhelm the anti-proteolytic defenses. Inflammatory cell and consequently endothelial cell proteases lead to the breakdown of pulmonary matrix and alveolar walls following particle inhalation, creating airspace enlargement and minimizing surface area for gas exchange (March et al., 2000). This protease theory is based on the 1963 finding that patients deficient in α-1 antitrypsin, a major neutrophil elastase inhibitor, have accelerated emphysema development (Laurell and Eriksson, 1963). The capability of proteases to cause emphysema was then verified by instilling them intratracheally (Gross et al., 1965; Janoff et al., 1977; Snider et al., 1984). Protease inhibition has since been tested in the animal model with varying efficacies, depending on the protease depleted. Serine proteases from neutrophils (59% protection in neutrophil elastase deficient mice) (Shapiro et al., 2003) as well as macrophage metalloproteases (Hautamaki et al., 1997) have been considered most important in a cigarette smoke induced disease model, but the more broad application of anti-inflammatory interventions has proven useful in smoked rodents as well (Churg et al., 2008). This is especially of interest, as sampling of human COPD specimens noted T lymphocytes in alveolar destruction and airflow obstruction, though smoking history was not assessed in the patient population (Hogg et al., 2004). This has shed light on the participation of auto-immunity contributing to the overall pathogenesis of emphysema through anti-endothelial cell antibodies and pathogenic CD4+ T lymphocytes (Taraseviciene-Stewart et al., 2005). Because emphysema was initially believed to occur through the same mechanism as SAR and to be the underlying cause of pulmonary hypertension, both in COPD as well as following cigarette smoke exposure, many studies have focused only on the emphysematous pathology, especially on the proteolytic aspect, neglecting further complex contributing factors.

## Small airway remodeling (SAR)

Emphysema is not the only manifestation of cigarette smoke induced COPD. SAR is also a major contributor to the disease as it limits airflow (Hogg et al., 2004) and is characterized by continuous repair for the duration of smoke exposure (Churg and Wright, 2009). While emphysema is the breakdown of extracellular matrix in the lung parenchyma, SAR pathogenesis involves a fibrotic process leading to airway narrowing due to airway wall thickening. Therefore, it is not surprising that SAR appears to develop through a mechanism independent of the one that drives emphysema. This correlates well with the fact that methods for emphysema prevention cannot be uniformly applied to treat SAR. Only TNF-α and IL-1 receptor knockouts (Churg et al., 2009b) as well as mice treated with an MMP-9/MMP-12 inhibitor (Churg et al., 2007a) were protected against the development of both emphysema and SAR when exposed to cigarette smoke, while other interventions such as chemokine receptor 5 (CCR5) (Bracke et al., 2007) and CCR6 (Bracke et al., 2006) knockouts are merely protected from emphysema following cigarette smoke.

## Pulmonary hypertension

COPD morbidity and mortality are significantly increased by the not uncommon occurrence of pulmonary hypertension associated with cigarette smoke (Chaouat et al., 2008; Elwing and Panos, 2008), which correlates with a poor prognosis (Weitzenblum et al., 1981). Originally, it was believed that the increase in pulmonary artery pressure is secondary to the lung pathology associated with COPD, as a result of hypoxia, emphysema, and loss of the vascular bed (Wright et al., 2005). With the realization that smoke and its mediators effect vasculature to induce remodeling, and that pulmonary hypertension is an early symptom that manifests long before airflow obstruction, emphysema, and SAR, the independent mechanism became evident (Wright et al., 2005). It has recently been shown that inducible nitric oxide synthase (iNOS) is important in the development of pulmonary hypertension following cigarette smoke exposure (Gielis et al., 2011; Kratzer et al., 2013). Mice lacking iNOS or treated with an iNOS inhibitor are protected against the cigarette smoke induced development of emphysema and pulmonary hypertension (Nathan, 2011; Seimetz et al., 2011). Both prostacyclin and endothelial NOS are protective against pulmonary hypertension induced by hypoxia, while endothelin-1 and VEGF contribute to the pathogenesis (Wright et al., 2004, 2005; Voelkel, 2008; Wright and Churg, 2008). The oxidative damage to the vasculature results from reactive nitrogen species that are not of endothelial source, but more likely contained in the cigarette smoke itself (Wright et al., 2012). The result is altered vasoconstriction and vasodilation as well as vascular cell proliferation leading to the pathological thickening of vessels. In the intima, elastin and collagen are deposited, while smooth muscle cells proliferate (muscularization), and adventitia harbors increased numbers of CD8+ T lymphocytes (Wright et al., 1983, 1992; Barbera et al., 2003).

Smoking is not only the main risk factor for COPD and pulmonary hypertension, but also for coronary artery disease summarized in a recent review (Ghoorah et al., 2012).

## The proteolytic and inflammatory response in COPD

COPD, like asthma, is characterized by chronic and abnormal inflammation of the distal airways resulting in airflow limitation, but differs from asthma in that it is progressive and largely irreversible. This chronic disease encompasses bronchiolitis and fibrosis with obstruction of small airways, vascular remodeling leading to pulmonary hypertension, destruction of lung parenchyma with loss of the alveolar wall and subsequent airspace enlargement defined as emphysema, as well as loss of lung elasticity. The underlying mechanisms involve protease/anti-protease imbalance following inflammation, with subsequent proteolytic matrix destruction (Churg et al., 2008), oxidant damage that leads to apoptosis of resident lung structure cells (Yoshida and Tuder, 2007; Churg et al., 2011). accelerated aging (Csiszar et al., 2009), a failure to repair, and the contribution of autoimmunity (Taraseviciene-Stewart et al., 2006; Maeno et al., 2007; Stampfli and Anderson, 2009). The most prominent and frequent etiology of COPD remains cigarette smoke and its onset is mid-life. For COPD, the inflammatory response is slowly progressive and leads to the actual destruction of lung parenchyma where alveolar walls disappear and the distal airspaces become pathologically and permanently enlarged, resulting in emphysema (Barnes, 2004). The contributing inflammatory cells in COPD are neutrophilic granulocytes, alveolar macrophages, and CD8+ T lymphocytes (Taraseviciene-Stewart and Voelkel, 2008; Stampfli and Anderson, 2009). The accumulation of both T and B lymphocytes following apoptosis of resident lung cells has been described to create follicles within the lung parenchyma, adjacent to airways, in COPD (Hogg et al., 2004; Taraseviciene-Stewart et al., 2006). In a rat model, neutrophil depletion did not prevent smoke induced emphysema, while treatment with anti-monocyte/macrophage antibody did. These findings implicate macrophages rather than neutrophils as the critical pathogenic factor in cigarette smoke induced emphysema (Ofulue and Ko, 1999). Macrophages are known to secrete the cytokines interleukin 8 (IL-8) and tumor necrosis factor alpha (TNF-α) as well as leukotriene B4 (LTB4), all found to be increased in COPD patients (Keatings et al., 1996). Additionally, macrophages also generate reactive oxygen species (ROS), monocyte chemotactic protein 1 (MCP-1) and elastolytic enzymes such as matrix metalloproteinase (MMP)-2, MMP-9, MMP-12, and cathepsins K, L, and S thus contributing to the lung destruction (Barnes et al., 2003).

Neutrophils are found to be increased in bronchial biopsies and sputum of patients during COPD, correlating with severity (Keatings et al., 1996; Di Stefano et al., 1998; Pesci et al., 1998; Retamales et al., 2001). Neutrophils apparently contribute to the disease pathogenesis by secreting serine proteases (neutrophil elastase, cathepsin G, proteinase) and metalloelastases MMP-8 and MMP-9 (Barnes et al., 2003).

The major pathogenetic factor of neutrophil and macrophage accumulation in emphysema is their secretion of proteases and inflammatory mediators. Cleaved fragments of elastin, collagen, and fibronectin are believed to possibly act as auto-antibodies, which would explain the accumulation of T and B lymphocytes in COPD patients (Hogg et al., 2004; Taraseviciene-Stewart and Voelkel, 2008), It has been shown that antibodies against endothelial cells correlate with alveolar septal cell apoptosis as well as the activation of MMP-2 and MMP-9, thereby initiating the proteolytic cascade and inducing emphysema independent of cigarette smoke (Taraseviciene-Stewart et al., 2005). This specific mechanism of anti-endothelial cell auto-immunity is dependent on CD4+ T lymphocytes believed to be pathogenic (Voelkel and Taraseviciene-Stewart, 2005; Taraseviciene-Stewart et al., 2007). Anti-endothelial cell antibodies were also detected in a rat model of cigarette induced emphysema (Taraseviciene-Stewart, unpublished observation).

## Genetic predispositions for COPD development

Interestingly, only 15–20% of smokers are susceptible to developing COPD, underlining the contribution of genetic factors (Fletcher and Peto, 1977). This susceptibility is mirrored in the animal model, where the development of cigarette smoke induced emphysema is strain-dependent (Guerassimov et al., 2004; Bartalesi et al., 2005). To this date, though, the only human genetic predisposition identified is the rare hereditary deficiency in α-1 antitrypsin (Laurell and Eriksson, 1963). Transgenic mice over-expressing genes of interest or knockout mice lacking a specific gene are exposed to mainstream or SHS elucidating how a specific gene modifies the pathogenesis of this disease. Though mice are so small that the assessment of disease progression and COPD symptoms is hindered, the application of transgenic and knockout mice to define the effects of a gene insertion or expression increase as well as that of a gene deletion, can answer many questions. Additionally, 80% of the mouse genome contains genes that have direct orthologues in the human genome and less than 300 genes (1%) are unique to the murine species (Waterston et al., 2002; Pennacchio, 2003). Interestingly, 89–90% of rat genes contain single orthologues in the human genome and 76% of well-characterized human disease genes are found in the rat genome (Gibbs et al., 2004). Both rats (Pauwels et al., 1985; Martin et al., 1992) and mice (Guerassimov et al., 2004; Bartalesi et al., 2005) have strain-dependent susceptibilities to excessive airway inflammation and smoke-induced emphysema, respectively, just as humans have genetic susceptibility to developing COPD.

## Preventative therapies

The most prominent interventions studied for COPD are anti-proteolytic, anti-inflammatory, as well as anti-oxidant and are directed against the development of emphysema, often not even being tested for SAR or pulmonary hypertension efficacies (Churg et al., 2011). Proteases are released by both accumulating inflammatory as well as resident cells, causing matrix destruction in the alveolar wall and therefore emphysema. This hypothesis is summarized in the term protease/anti-protease imbalance, because both an excessive protease release as well as a hindered anti-protease activity is necessary to contribute to the pathogenesis. The inhibition of various proteases during cigarette smoke exposure has been tested: serine proteases (neutrophil elastase) (Cavarra et al., 2001; Takubo et al., 2002; Wright et al., 2002; Churg et al., 2003b; Shapiro et al., 2003; Pemberton et al., 2006), different metalloproteases (MMP-9 and MMP-12) (Hautamaki et al., 1997; Selman et al., 2003; Mahadeva and Shapiro, 2005; Pemberton et al., 2005; Churg et al., 2007a), and cysteine proteases (cathepsins B and S) (Kang et al., 2007). The preventative success depends on the specific protease inhibited.

The immune reaction to chronic smoke exposure has been investigated by blocking certain inflammatory responses using knockout mice or simply applying an anti-inflammatory treatment. Both anti-TNF-α receptor (Churg et al., 2004; D'Hulst et al., 2006) and anti-PDE4 (Rofluminlast) (Martorana et al., 2005) therapies are protective anti-inflammatory measures in the animal model, though these results could not be as successfully reproduced for the chronic human disease (Barnes, 2007). Significant protection against SHS induced emphysema in the animal model was also achieved using a hyaluronan aerosol (Cantor et al., 2005) and by blocking interferon γ (INF-γ) or CCR5 (Ma et al., 2005; Bracke et al., 2007) or CCR6 (Bracke et al., 2006), After it was shown that SCID (severe combined immuno-deficient) and Rag (recombinase activating gene) knockout mice, which both lack functional T and B lymphocytes, are not protected from mainstream smoke induced emphysema (D'Hulst et al., 2005a), CD4+ and CD8+ T lymphocyte-deficient mice were tested individually. While CD4+ T lymphocyte-defecient mice are not at all protected, CD8+ T lymphocyte-deficient mice are 100% protected against emphysema following mainstream exposure (Maeno et al., 2007). Toll-like receptor 4 (TLR4) knockout mice were also not protected (Maes et al., 2006). Statins, however, have proven to be protective against cigarette smoke induced emphysema in rats (Lee et al., 2005) and appear to be promising therapy in humans as well (Soyseth et al., 2007). Simvastatin additionally is effective against pulmonary hypertension, but unfortunately does not prevent SAR (Wright et al., 2011), unless administered before disease onset (Lee et al., 2005; Ou et al., 2009).

ROS are both contained in cigarette smoke and released by the resultant infiltrating cells, such as neutrophils and macrophages (Pryor and Stone, 1993; Rahman and MacNee, 1996; MacNee, 2001). This, again, leads to an imbalance, that of oxidants and anti-oxidants, so that the lung is exposed to oxidative stress (Petrache et al., 2006). Susceptibility to oxidative stress is, like the genetic predisposition for cigarette smoke induced emphysema, specific to a certain strain of mice (Cavarra et al., 2001; Bartalesi et al., 2005). Oxidant sensitive mice show less anti-oxidant capacity when exposed to cigarette smoke, developing emphysema. This effect can be reduced when applying an oral anti-oxidant (Koul et al., 2003), while elimination of the redox sensitive transcription gene Nuclear factor 2 (Nrf2) enhanced the cigarette smoke induced oxidative stress and emphysema in the otherwise resistant ICR mouse strain (Rangasamy et al., 2004).

Concisely, emphysema induced by cigarette smoke is a multi-factorial disease and therapeutic approaches should be undertaken to repair the lung structure by preventing marix degradation by reducing proteases, inflammation, oxidative stress, cell death, and/or autoimmune-mediated destruction.

## Study parameters thus far

Here we have compiled 114 publications containing 155 studies addressing cigarette smoke exposure in the animal model. Approximately half of the studies (71 experiments) are mainstream exposure (Table 1). Side stream smoke exposures (Table 2) make up 77 experiments of the studies listed herein and a small portion (7 studies) is unclear about the exposure method utilized (Table 3). Of the studies listed here, a large portion (115 studies) utilizes quite a strain diversity of mice (C57Bl/6, DBA/2, Balb/C, A/J, ICR, FVB, or a strain not described), although C57Bl/6 mice are dominant (83 experiments). C57Bl/6 mice are a common strain for knockout studies and generally a popular model for cigarette smoke induced emphysema (Takubo et al., 2002; Guerassimov et al., 2004; Bartalesi et al., 2005; Yao et al., 2008; Botelho et al., 2010) due to their deficiency in anti-elastase (Gardi et al., 1994; Cavarra et al., 2001). Balb/C mice with higher levels of the cytokine granulocyte-macrophage colony stimulating factor (GM-CSF) (Morris et al., 2008; Botelho et al., 2010, 2011b) and the oxidant sensitive strain DBA/2 (Cavarra et al., 2001; Bartalesi et al., 2005) have also been shown to have severe emphysema following cigarette smoke exposure. ICR mice are considered oxidant resistant and are of interest when attempting to induce disease (Cavarra et al., 2001; Rangasamy et al., 2004; Hodge-Bell et al., 2007; Yao et al., 2008). A/J express less severe pulmonary complications (Guerassimov et al., 2004; Yao et al., 2008; Braber et al., 2010) and have found use in drug tests, but have been used as a model for chronic cigarette smoke exposure as well (Foronjy et al., 2005; March et al., 2005, 2006). Apart from mice, one study addresses rabbits, 15 utilize guinea pigs (either Hartley strain or not named), while 24 studies gravitate toward using rats. These are almost exclusively Sprague-Dawley, while spontaneously hypertensive rats (SH) as well as Wistar Kyoto are also popular.

Originally, only studies from 1997 onwards were added to Tables 1–3, but with such consistency of certain researchers to repeatedly reference earlier publications, the original publishers of certain exposure procedures were included as far back as 1974. A number of authors stumble when it comes to actually stating clearly whether their exposure method was mainstream or side stream. This is most likely due to the assumption that this would be known according to the exposure system more or less detailed in the Materials and Methods section or merely mentioned in the abstract. The Materials and Methods section, though, frequently references a publication that, in turn, references the original design. Such a basic fact as mainstream or side stream exposure must be pointed out, as must the fact that there was a cigarette smoke exposure at all, also something that can be quite hidden in a less specific manuscript. Fortunately, many publications do announce a head/nose only or a whole body exposure in the Materials and Methods section, if mainstream or side stream (environmental or second hand) has not already been mentioned in the abstract or introduction. There are some that allow you to look up their chamber of choice, although this leads to lengthy product guidelines tediously researched online (i.e., the long outdated Hamburg II chamber). There are only a few groups that generally use the same procedure and one can at least assume that they are utilizing the same method, simply using their latest publication as the current reference (which then must be referenced back to the original procedure). It is a relatively safe assumption that the method always remains mainstream or side stream within the same research group, unless otherwise noted, but it is left for the reader to assume, nonetheless. In this summary, only two research groups resort to this form of documentation.

Seven publications segregated in Table 3 leave it completely unknown what exposure they have used, and one uses a “whole body mainstream” exposure, apparently employing a high concentration of cigarette particulates into a chamber to mimic mainstream smoking (Braber et al., 2011), but that is, again, left to be assumed. Generally, one can still assess the exposure method in most cases, it becomes less clear at the level of nicotine, carbon monoxide, and particulate levels. Some researches are very diligent here, while others may not have measured these parameters and therefore do not address them. There is also somewhat variability to whether nicotine and TSP/TPM are measured as mg/m^3^ or μg/l (which can easily be calculated to mg/m^3^). Some quote mg/cig for the nicotine levels (Pittilo et al., 1982, 1990; Suemaru et al., 1992; Escolar et al., 1995; Wright and Sun, 1999; Wright et al., 1999; Balansky et al., 2000; Cavarra et al., 2001; Wright and Churg, 2002; Rangasamy et al., 2004; Tanaka et al., 2004; Bartalesi et al., 2005; Martorana et al., 2005; Yao et al., 2008; Woodruff et al., 2009; Cicko et al., 2010; Miller et al., 2010; Yoshida et al., 2010; Nie et al., 2012; Nemmar et al., 2013), meaning this was likely looked up through the manufacturer. The same is true for carbon monoxide, which is either measured in ppm or percentage of carboxyhemoglobin in the serum. It is clear that the vast majority of studies has, at bare minimum, one of these parameters missing, as only 24 (15.5%) exemplary studies listed all three. Usually, the levels named are an average kept during the entire exposure period, which is of importance when considering SHS exposure. The TSP/TPM levels in SHS exposures is as low as 24 mg/m^3^ using a unique rabbit model (Sun et al., 2001), but as high as 250 mg/m^3^ in standard Teague chamber mouse models using Kentucky reference cigarettes (Foronjy et al., 2005, 2006; Golovatch et al., 2009; Richens et al., 2009). The Stampfli lab (Botelho et al., 2010, 2011a,b, 2012; Farkas et al., 2011) and one other group (Braber et al., 2010) go beyond 700 mg/m^3^ for their mouse models that do not utilize the Teague chamber.

There is still diversity in the use of cigarettes (Table 4), despite the fact that reference cigarettes, specifically designed for this type of research, have been available for approximately four decades now. The vast majority of studies (111 studies making up 71.6%) do use the reference cigarettes provided by the University of Kentucky's Tobacco Research and Development Center (formerly the Tobacco and Health Research Institute). Two groups have listed all necessary parameters with the unique cigarettes (Ultratech Corp and Ye Shu) they have chosen (Sun et al., 2001; Nie et al., 2012). Unfortunately, it appears that commercial Virginia cigarettes are popular, without specifying which kind, though apparently generally a type with 0.9 mg nicotine per cigarette, except for one study that claims to use Virginia cigarettes with 1 g nicotine per cigarette, presumably a typo (Escolar et al., 1995). A total of 17 studies do not list the cigarettes used (either commercial unfiltered, simply unfiltered, or no reference at all), while five publications compromising eight studies do opt for Kentucky reference cigarettes, but then neglected to list which kind, let alone the parameters (Hautamaki et al., 1997; Shapiro et al., 2003; Maeno et al., 2007; Nakanishi et al., 2009; Clauss et al., 2011).

Other cigarette brands listed in Tables 1–3 stated only some or none of the parameters for TSP/TPM, nicotine, and carbon monoxide levels. The eight remaining brands of cigarettes are Marlboro 100 and Marlboro red (for Marlboro Medium sort, the nicotine level is 1 mg/cig and carbon monoxide is 12 mg/cig), Long Peace and Seven Star (Japan), Eighty Eight Lights (South Korea), Arda-Bulgartabac (Bulgaria), Canada Tobacco, and Virginia brand. For these cigarettes, the parameters could not be found to supplement the information available in the original publications themselves, as the actual type of cigarette was not stated in any one of them. Therefore, data regarding reference cigarettes used in the studies summarized here have been left incomplete in Table 4.

Kentucky reference cigarettes, on the other hand, are very well documented, but sometimes it is unclear in a publication whether a mix of two types was used for smoke exposure. At times, the filtered version (named with an “F” by the manufacturer) is used, but the filter is removed. There are also those who list using 2R4, which presumably means that the filter was removed for the study. The first line of reference cigarettes from the University of Kentucky were from 1974 and included 1R3, 1R3F, and 2R1, of which the filtered kind (1R3F) had 30 mm butts and the unfiltered cigarettes (1R3 and 2R1) could be purchased with either 23 or 30 mm butt lengths. In 1983, the new generation of reference cigarettes was 1R4F, only available in a 35 mm butt length. In 2001, the next generation, named 2R4F, was launched and as of 2008 3R4F has been produced. Studies presumably reflect the reference cigarette of the time and the well-documented levels of Kentucky reference cigarettes can be found in their entirety in Table 4.

## Comparing mainstream and second hand exposures

Although parameters appear to rarely be listed to the extent they should be, we have attempted to compare the direct exposure of smoke in these animal studies to those of animals exposed via a whole body method, mimicking SHS. One must remember that while mainstream smoke is inhaled into the lungs from the cigarette directly, SHS is the inhalation of suspended particles from the smoker's exhale and the burning end of the cigarette, thereby concentrations of particles and individual components are not necessarily reduced, as one would hope. What becomes clear is, that SHS exposure does indeed lead to emphysema and other COPD symptoms in animal models. A multitude of studies elucidate this fact and they are summarized in Table 2, which also highlights what effects (whether emphysema, SAR, or pulmonary hypertension) were assessed.

Studies to unravel the pathogenic mechanisms of cigarette smoke exposure were undertaken in the knockout models, where the effect can easily be seen based on only one factor. The central role of TNF-α has been documented for both exposure applications. This was made evident by inhibition of TNF-α using a receptor knockout model (TNFR KO), which attenuated the development of emphysema by 71–83% and of SAR by 100% in mainstream cigarette smoke exposure (Churg et al., 2002a, 2004, 2009b). TNFR KO were 100% protected against emphysema using side stream smoke (D'Hulst et al., 2006). Additionally, inhibition of efferocytosis is TNF-α dependent in the side stream knockout model (Richens et al., 2009).

C–C CCR5 knockout mice were 100% protected against emphysema in an exposure method that was not defined, where INF-γ over-expression enhanced emphysema (Ma et al., 2005). In a side stream exposure study (Bracke et al., 2007), the effect of CCR5 knockout was only protective to 25%, whereas CCR6 knockout was more effective with 67% protection against emphysema (Bracke et al., 2006). It is possible that the same manipulation would be more protective in a mainstream versus a second hand exposure, although the exposure in the first study, which assessed a number of pathways leading to apoptosis, is not known.

Another well-documented knockout model for the mechanism of smoke induced pathology is that of macrophage elastases, specifically MMP-12 (Hautamaki et al., 1997; Churg et al., 2002a, 2003a; Shapiro et al., 2003) and MMP-9 (Atkinson et al., 2010). The elastinolytic activity central to matrix breakdown and alveolar enlargement had already been attributed to macrophages (Ofulue et al., 1998; Ofulue and Ko, 1999) and these two enzymes were pinpointed via inhibition (Churg et al., 2007a). Here, MMP-12 (also termed macrophage metalloelastase MME) has been identified to be of foremost importance in lung destruction, since MMP-12 knockout mice are resistant to smoke induced emphysema, while emphysema can occur independent of MMP-9 (Atkinson et al., 2010). Mice deficient in monocytes and macrophages do not develop enlarged airspaces upon cigarette smoke exposure, while those deficient in polymorphonuclear cells (PMN) do (Ofulue et al., 1998; Ofulue and Ko, 1999; Dhami et al., 2000). Unfortunately, all studies regarding MMP contributions, which are considerable, have solely been tested in the mainstream smoke model.

The neutrophil-derived serine elastase has been implicated in both a mainstream and a side stream exposure model (Dhami et al., 2000). In a direct smoke exposure model, the neutrophil elastase knockout mouse is 59% protected against emphysema development (Shapiro et al., 2003), while specific neutrophil elastase inhibitors are similarly protective in the guinea pig model (Wright et al., 2002; Stevens et al., 2011). In a SHS exposure model of the mouse, one group (Stevens et al., 2011) was also able to detect a marked decrease in SAR development in both their mainstream and side stream exposure models upon treatment with neutrophil elastase inhibitor AZD9668.

The only thus far defined true genetic factor described in man predisposing to COPD is α-1 antitrypsin deficiency. This deficiency leads to accelerated development of emphysema following cigarette smoke exposure. Although the importance of α-1 antitrypsin has been documented in the animal model of direct cigarette smoke (Dhami et al., 2000; Churg et al., 2003b), there are two studies where it is not clear whether an indirect exposure was utilized (Takubo et al., 2002; Churg et al., 2007b). Nevertheless, α-1 antitrypsin is indeed protective against emphysema in a dose-dependent manner, indicating that this is likely also true for indirect cigarette smoke exposure (Dhami et al., 2000; Churg et al., 2003b).

In addition to genetic manipulations elucidating molecular mechanisms of cigarette smoke pathogenesis, there are also models for drug testing, more specific to an intervention protocol. These go beyond simply attenuating the proteolytic breakdown via inhibition of just one protease and encompass mainly statins and anti-oxidants. Simvastatin, as mentioned above, has a protective effect against emphysema and pulmonary hypertension, but not SAR, and has been tested in both the guinea pig (Wright et al., 2011) and the rat (Lee et al., 2005), but only using a mainstream exposure method. Therefore, a homologous mechanism for side stream smoke is yet to be described.

Beyond being influenced by mouse strain (Guerassimov et al., 2004; Bartalesi et al., 2005; Foronjy et al., 2005), emphysema development is also dependent on the airspace content of anti-oxidants. This was demonstrated by comparing bronchoalveolar lavage (BAL) levels of endogenous anti-oxidants from the oxidant resistant mouse strain ICR with that from other strains (Cavarra et al., 2001; Richens et al., 2009). Successful anti-oxidant therapy is seen for mainstream (March et al., 2006; Suzuki et al., 2009; Churg et al., 2012) and side stream exposure models (Smith et al., 2002; Richens et al., 2009), as well as in a Rtp801 knockout model for side stream smoke (Yoshida et al., 2010). This is reflected in the success of superoxide dismutase (SOD) transgenic mice (Foronjy et al., 2006; Richens et al., 2009), specifically those with enhanced SOD3 expression (Yao et al., 2010), in protecting against emphysema in both exposure scenarios.

## What is missing

Unfortunately, even with chronic exposure studies, the animal model has limitations to how well it can mimic COPD, as seen by the inadequacy of interventions that protect mice against cigarette smoke induced emphysema, but not humans (Barnes, 2007). A procedure that is applied in the same way for each study could greatly contribute to our understanding of pathogenesis and how the animal model relates to the human disease. For example, with a standard 6-month exposure period for mouse models, research groups have been able to establish a disease time frame that is utilized for most chronic studies. In rats, this chronic period starts at 2 months of exposure (Ofulue et al., 1998; Kratzer et al., 2013) and in guinea pigs after 3 months of exposure (Wright et al., 2002). In order to truly compare results, though, it is of importance to standardize exposure methods and levels as well.

Generally, there is favoritism toward the somewhat more recent application of chambers, which was greatly improved so as to be utilized as a standard method for SHS exposure in 1994 (Teague et al., 1994). A chamber may contain any number of animals and is often applied to produce a cigarette smoke mixture consisting of 89% side stream and 11% mainstream smoke, exposing animals to environmental smoke versus attaching a nose or head piece to introduce smoke first hand. Often, though, even mainstream or side stream smoke exposure are neglected to be pointed out, as it is assumed that using a chamber method should suffice as information, even though it can require extensive back-referencing to discover the actual original procedure. Beyond the smoke chamber, which wasn't developed until 1994 (Teague et al., 1994), few standardized procedures were possible. Since then, though, there is still very little homogeneity in the applications used for research in this field. We could greatly improve our understanding of the consequences of inhaled cigarette smoke if we were to apply a similar and comparable model in every case. This would require selecting conform research cigarettes, whether filtered or not, preferably from the same source, so that contents and levels of important toxins and mediators can be compared.

If a variety of cigarettes are necessary to accommodate research across the globe, it is imperative that at least cigarette parameters are assessed and noted. Reference cigarettes were specifically designed from 1969 onwards for the purpose homogenous and comparable studies around the world. To this day, commercial cigarettes with varying or unknown parameters are applied.

There is still an abundance of publications that do not mention or may even not determine nicotine or carbon monoxide amounts or the contributing particles in cigarettes (whether as TSP or TPM), though it could easily be evaluated from online supplements for major cigarettes in use. For randomly selected commercial cigarettes, of course, there is not even this option, as only nicotine and in very few cases carbon monoxide levels are available. Some studies do not even list the cigarette type or brand used, so there is practically no information concerning the exposure. These factors complicate matters even further, as there are already numerous ways that cigarette smoke is administered, using as few as two cigarettes a day in mice (Hautamaki et al., 1997; Shapiro et al., 2003) or as many as 12 cigarettes a day in rats (Nie et al., 2012).

Additionally, many studies use an initial acclimation period for their cigarette smoke exposure, employing an increasing number of cigarettes or longer smoke periods after beginning the study, thereby also allowing variability, but acclimation is usually necessary. Inconsistencies can be avoided by assessing the most important parameters, such as nicotine, carbon monoxide, and particle levels. If the scientific community could agree on a protocol to establish similar exposures both for mainstream and side stream cigarette smoke, results would be much more comparable. At a bare minimum, simply correctly documenting the method used would add a great deal to the knowledge that could have already been gained. In this way, cigarette smoke researchers all around the world would be able to collectively assess and compare their data.

## Conclusion

Cigarette smoke exposure is the most important risk factor for developing COPD, both in smokers and in non-smokers who are involuntarily exposed to the toxic and carcinogenic contents of environmental cigarette smoke. Studies of long-term cigarette smoke exposure effects are very costly, time-consuming, and require an abundance of resources. For this reason, tests should be optimized so that experimental models are comparable and provide as much knowledge as possible. It is evident from the literature that there is, to this day, enormous unnecessary diversity in study designs despite improvements to our experimental exposure methods (chambers and reference cigarettes). There is a great need for a standard protocol defining parameters to be evaluated and exposure procedures to be followed. With this quite simple documentation of easily assessed facts and we would be able to gain much more knowledge with each study. It should be possible to determine molecular mechanisms, oxidative stress, protease participation, inflammatory cytokine levels, and cellular death following mainstream and side stream cigarette smoke exposure, comparing them. These insights would greatly improve and expand today's comprehension of cellular and molecular mechanisms implicated in the pathology of COPD. A more defining knowledge of the pathogenesis of mainstream and second hand cigarette smoke induced emphysema, SAR, and pulmonary hypertension could have been achieved with the undertaken studies if a unified procedural method had been applied.

Despite the shortcomings in terms of documentation in a large amount of experiments, they have nonetheless led to great advances through gene knockout models or transgenic mice as well as inhibitory tactics and drug treatments. When combining these animal studies in one overview, it becomes highly evident that second hand cigarette smoke has many of the same mechanisms and detrimental effects that mainstream smoke does. For this reason, we wish to point out that the US Surgeon General's assessment of a missing link should be revisited, so that the public becomes more aware of the health risks to innocent bystanders, where SHS exposure causes COPD/emphysema in non-smokers.

## Funding

Funded by AHA 0735388N, 11GRNT7520020, FAMRI CIA 072053, Emphysema Research Fund and Bixler Family Foundation.

### Conflict of interest statement

The authors declare that the research was conducted in the absence of any commercial or financial relationships that could be construed as a potential conflict of interest.
